# Advances in imaging and artificial intelligence for precision diagnosis and biopsy guidance in prostate cancer

**DOI:** 10.3389/fonc.2025.1614891

**Published:** 2025-10-13

**Authors:** Ye Wu, Qiang Lu, Zhifu Liu, Jianhe Wu, Xianya He, Yongjun Yang, Yuanwei Li

**Affiliations:** Department of Urology, The First Affiliated Hospital of Hunan Normal University/Hunan Provincial People’s Hospital, Changsha, Hunan, China

**Keywords:** artificial intelligence, image technology, multimodal image fusion, prostate biopsy, research progress

## Abstract

Early and accurate diagnosis of prostate cancer is critical for optimizing patient prognosis. However, traditional transrectal ultrasound-guided systematic biopsy (TRUS-Bx) has a relatively high false-negative rate. This is attributed to limitations such as insufficient anatomical coverage and inadequate assessment of tumor heterogeneity. Multiparametric magnetic resonance imaging (mpMRI), when combined with the Prostate Imaging Reporting and Data System (PI-RADS), has substantially improved the diagnostic specificity of clinically significant prostate cancer (csPCa; Gleason grade ≥ 3 + 4). Nevertheless, its discriminatory ability for PI-RADS 3 lesions remains restricted. In recent years, multimodal image fusion technology has boosted the detection rate of csPCa by 10%-15% via precise lesion localization. Molecular imaging exhibits a sensitivity of up to 95% (range: 90-98%) in the whole-body staging of high-risk patients, particularly for nodal metastases. Artificial intelligence (AI), through deep-learning algorithms, optimizes lesion segmentation and image texture analysis, thereby significantly enhancing the detection rate of csPCa in targeted biopsies. Looking ahead, it is essential to integrate multimodal imaging and genomic data, construct individualized risk-stratification models, and facilitate the clinical translation of low-cost and standardized technologies. This article comprehensively examines the synergistic mechanisms of imaging and AI technologies in the diagnosis and biopsy guidance of prostate cancer, offering a theoretical foundation for precision medicine practice.

## 1 Introduction

PCa is the second most prevalent malignant tumor among men globally, accounting for approximately 14.2% (1). Early and accurate diagnosis of prostate cancer is a pivotal aspect in enhancing prognosis (2). At present, prostate biopsy remains the “gold standard” for diagnosing prostate cancer. However, traditional systematic biopsy techniques are characterized by both a high false-negative rate and overdiagnosis, defined as the detection of indolent cancers that may not progress, potentially leading to unnecessary treatment. This is due to insufficient anatomical coverage and limitations in evaluating tumor heterogeneity. Additionally, these techniques are associated with risks such as infection and bleeding (2). Notably, for patients under active surveillance, false-negative results may delay the treatment opportunity and increase the reclassification risk (10%-25%) (3).

The advent of multiparametric magnetic resonance imaging (mpMRI) represents a significant breakthrough in image-guided techniques. When integrated with the PI-RADS, it can substantially improve the diagnostic specificity of clinically significant prostate cancer (csPCa, Gleason grade ≥ 3 + 4) to approximately 0.83 (range: 0.76-0.89) in high-risk cohorts, compared to historical values around 0.248 (4). However, mpMRI has restricted discriminatory capacity for PI-RADS 3 lesions, with a false-negative rate of 20% (2). Consequently, multimodal image fusion technology has substantially optimized the detection efficiency through precise lesion localization. Molecular imaging modalities, such as prostate- specific membrane antigen positron emission tomography/computed tomography (PSMA PET/CT), through the quantification of tumor metabolic heterogeneity, provide high-sensitivity support for the staging of high-risk patients (5). Moreover, artificial intelligence-driven image analysis techniques, by integrating multimodal data (radiomics, genomics, clinical parameters), construct individualized prediction models, gradually attaining optimization of the entire process from diagnosis to prognosis (6).

This article comprehensively reviews the progress in the application of imaging techniques and artificial intelligence in prostate biopsy, analyzes their synergistic mechanisms, and explores the future directions of development.

Contribution of this review: This narrative review synthesizes the latest evidence on imaging and AI technologies in prostate biopsy, highlights their synergistic mechanisms, and proposes future directions for integrating multimodal data into clinical practice. It serves as a comprehensive reference for urologists and radiologists seeking to implement precision biopsy strategies.

## 2 Limitations of traditional prostate biopsy techniques

### 2.1 Traditional technique: transrectal ultrasound-guided systematic biopsy

The cornerstone of traditional prostate biopsy is transrectal ultrasound-guided systematic biopsy (TRUS-Bx). This approach utilizes transrectal ultrasound to localize the prostate and, following a standardized protocol, systematically samples from regions such as the peripheral zone and the middle lobe of the prostate (typically with 10–12 needles). The objective is to cover the potentially cancer-prone areas of the prostate through randomly distributed tissue cores (7). For a long time, its ease of operation and relatively low cost have led to its being regarded as the “gold standard” for prostate cancer diagnosis (7). Nevertheless, as clinical evidence has accumulated, the limitations of TRUS-Bx have gradually become more pronounced. Traditional systematic biopsy is associated with a high false-negative rate (10–30% for csPCa) and overdiagnosis, defined as the detection of indolent cancers (e.g., Gleason score 6) that may not progress, potentially leading to unnecessary treatment. In contrast, oversampling refers to excessive biopsy cores that do not improve detection. These rates vary by clinical context (e.g., biopsy-naïve vs. repeat biopsy, transrectal vs. transperineal approach) (8–10). The limitations are primarily manifested in three aspects: inadequate anatomical coverage, limited detection efficiency, and suboptimal assessment of tumor heterogeneity.

### 2.2 Limitation analysis

#### 2.2.1 Insufficient anatomical coverage and lesion missed diagnosis

Prostate-specific studies have quantified the risk of missing anterior tumors. In patients with low-risk prostate cancer, up to 16% of csPCa lesions are located in the anterior part and are frequently missed by standard TRUS-Bx templates (11). Importantly, this risk is context-dependent. While expanding the biopsy scope to include anterior sampling can increase the detection rate of csPCa, the net benefit varies. For instance, in the biopsy-naïve population, anterior sampling may increase the detection rate of csPCa by approximately 5.7% (p=0.09, not statistically significant in that cohort), whereas in men on active surveillance (AS) with prior negative biopsies, the incremental yield can be higher, underscoring the need for risk-adapted sampling strategies (11).

#### 2.2.2 Bottleneck in the detection rate of random sampling

Traditional techniques rely on random sampling, which makes it arduous to effectively detect tumors that are small in volume (<0.5 cm³) or have an atypical distribution. The random sampling nature of TRUS-Bx creates a significant detection bottleneck, particularly for small or atypically located tumors. The false-negative rate is not uniform across all patient groups. For example, in patients under AS, a 12-core systematic biopsy may miss approximately 10% of csPCa (12). Notably, the reclassification risk following a negative biopsy is a key metric of this limitation. Cohort studies focused on AS populations report that patients with an initial negative biopsy harbor a 10%-25% risk of being reclassified to higher-risk disease upon subsequent surveillance biopsies, a figure directly attributable to sampling error and tumor multifocality (13). This phenomenon is closely associated with the multifocality and spatial heterogeneity of tumors (13).

#### 2.2.3 Inadequate assessment of tumor biological heterogeneity

The limited number of biopsy samples (usually 12 cores) may not comprehensively represent the genomic diversity of tumors. For instance, the correlation between the genomic risk score of low-risk patients and postoperative pathological upgrading and biochemical recurrence suggests that traditional biopsies may underestimate tumor aggressiveness (14). Furthermore, the Gleason scoring system’s disregard for minor Gleason 5 components can impact the accuracy of prognostic assessment (15).

#### 2.2.4 Operator dependence and standardization variations

The operator-dependent nature of TRUS-Bx is a well-documented limitation. Discrepancies in the definition of the ‘standard 12-core’ distribution among different institutions (e.g., inclusion of anterior or apical sampling) contribute significantly to inter-institutional variability in detection rates and limit result consistency (7). Furthermore, the reliance on ultrasound alone (resolution ~1–2 mm) for targeting is a fundamental constraint. Studies quantifying operator performance suggest that insufficient experience can reduce the detection rate of csPCa by a relative margin of up to 15-20% compared to expert operators, highlighting the critical impact of expertise on procedural efficacy (12).

### 2.3 Clinical impact and improvement directions

The aforementioned limitations directly influence the accuracy of clinical decision-making. Missed diagnosis of anterior cancer may misclassify low-risk patients as “benign” or “very low-risk,” thereby delaying the opportunity for radical treatment (11). False-negative results may extend the monitoring period, increasing patients’ psychological burden and the risk of complications associated with repeated biopsies (13). Misjudgment of tumor heterogeneity can interfere with the formulation of genomic risk stratification and personalized treatment decisions (such as the choice between AS and aggressive treatment) (14, 15).

To overcome the bottleneck of traditional techniques, targeted biopsy guided by new imaging techniques has significantly enhanced the detection rate of anterior and high-risk lesions through image-pathology fusion techniques (the detection rate of csPCa has increased by 10%-15%) (7, 12). Additionally, expanding the number of cores (such as 24-core extensive biopsy) combined with anterior sampling has also been shown to optimize the detection rate, but considerations such as an increased risk of infection need to be taken into account (11). In the future, it will be essential to further integrate imaging and genomic data to develop a precise sampling strategy for more individualized risk stratification and management. A comparative analysis of prostate biopsies that are guided by various imaging techniques is presented in **Table 1**.

**Table 1:** Comparative analysis of prostate biopsies guided by different imaging techniques.

Parameter	TRUS-Bx	Micro-US	MPMRI	PSMA PET/CT
Sensitivity (%)	40-50	75-90	73-95	89-98
Specificity (%)	60-70	65-80	76-97	82-99
Advantages	Simple operation Low cost	Real - time imaging Cost lower than MRI	High sensitivity for csPCa Reduces unnecessary punctures	Exceptional sensitivity for metastases Whole-body staging capability
Disadvantages	High false - negative rate High missed diagnosis rate Operator-dependent	Operator-dependent Limited depth penetration	Requires specialized equipment & expertise Higher cost Indeterminate PI-RADS 3 lesions	High cost Limited availability Lower sensitivity for low-grade (Gleason 6) tumors
Clinical Context & Notes	Primarily for systematic sampling in biopsy-naïve patients Per-patient analysis	Used as an MRI alternative for initial targeting Per-lesion analysis	Used for biopsy-naïve or prior-negative patients; based on PI-RADS scoring (per-lesion or per-patient)	Staging of intermediate/high-risk patients, biochemical recurrence, & metastasis detection Per-lesion analysis
References	(16–18)	(19–21)	(22–24)	(25–27)

## 3 Innovation and clinical application of imaging technologies

### 3.1 Ultrasound technologies: from conventional ultrasound to micro-ultrasound

TRUS-Bx is plagued by low sensitivity to early cancerous lesions. The advent of micro-ultrasound technology has substantially enhanced the detection capacity of blood flow signals.

#### 3.1.1 Super-microvascular imaging

Super-microvascular imaging (SMI) is capable of real-time visualization of abnormal microvessels within the prostate (such as tortuous and increased branching patterns), facilitating the identification of suspicious areas and guiding targeted biopsy. Studies have indicated that, in comparison to traditional color Doppler flow imaging (CDFI) and power Doppler ultrasound (PDUS), SMI exhibits a greater aptitude for detecting low-velocity blood flow and can more sensitively discern the neovascularization signals of tumors <mark>in the prostate (28). Prostate biopsy guided by SMI can significantly elevate the positive rate of tissue sampling (29). When integrated with other techniques like ultrasound elastography, SMI can dynamically monitor changes in blood flow signals, optimize the puncture trajectory, and mitigate accidental damage to normal blood vessels (30). SMI’s ability to display microcirculation without the need for contrast agent injection circumvents contrast-related risks, rendering it particularly suitable for patients with renal insufficiency.

#### 3.1.2 Contrast-enhanced ultrasound

Through the utilization of specific contrast agents, contrast-enhanced ultrasound (CEUS) can generate high-resolution images of tissue microvessels, enabling the observation of lesion characteristics, such as those of tumors and inflammations. Prostate cancer demonstrates rapid and high-intensity contrast enhancement attributed to neovascularization. CEUS can precisely delineate the tumor boundary and direct targeted biopsy (31, 32). Research reveals that targeted biopsy guided by CEUS has a 15%-20% higher cancer detection rate compared to systematic biopsy (31), while simultaneously reducing the number of unnecessary biopsies.

### 3.2 Magnetic resonance technologies: from biparametric to multiparametric

Biparametric magnetic resonance imaging (bpMRI) and mpMRI each possess distinct advantages in clinical diagnosis.

#### 3.2.1 Simplified efficacy of bpMRI

BpMRI encompasses only T2-weighted imaging (T2WI) and diffusion-weighted imaging (DWI)/apparent diffusion coefficient (ADC) sequences, excluding the dynamic contrast-enhanced (DCE) sequence. This not only substantially shortens the examination duration and reduces costs by approximately 50% but also eliminates risks associated with contrast agents (such as allergies, renal impairment, etc.) (33). Multiple meta-analyses indicate that the sensitivity (0.74-0.79 vs. 0.76-0.84) and specificity (0.88-0.90 vs. 0.89-0.89) of bpMRI and mpMRI are comparable, with no statistically significant difference (22, 23). Moreover, outside the prostate-specific antigen (PSA) range of 10–20 ng/ml, there is no marked difference in the cancer detection rates between bpMRI and mpMRI (24). Nevertheless, its sensitivity to certain lesions (such as tumors in the transition zone) may be lower than that of mpMRI, particularly when differentiating PI-RADS 3–4 lesions (33).

#### 3.2.2 Comprehensiveness of MpMRI

By integrating T2WI, DWI, DCE, and magnetic resonance spectroscopy (MRS), MpMRI can more comprehensively assess prostate lesions, including complex manifestations such as seminal vesicle invasion and pelvic lymph node metastasis. Studies demonstrate that the predictive accuracy of mpMRI for prostate extracapsular extension reaches 89%, significantly superior to traditional imaging (34). Its targeted biopsy (TB) in combination with ultrasound fusion exhibits higher sensitivity (81%-86%) and specificity (69%-84%) in the detection of csPCa, especially in lesions with PI-RADS ≥ 4, where its diagnostic efficacy is markedly better than that of traditional 12-core systematic biopsy (SB) (35–37). Combining TB and SB can further optimize the detection rate, particularly for multifocal lesions or in the anterior prostate region, with an incremental csPCa detection rate of 10%-15% (37–39). It is noteworthy that approximately 19%-31% of csPCa are detected solely in the second or third targeted biopsy, suggesting the necessity of a multi-core sampling strategy (36). Although several studies have shown that the sensitivity and specificity of bpMRI and mpMRI in detecting prostate cancer are marginally different, mpMRI demonstrates higher diagnostic accuracy in certain cases, especially in the detection of csPCa, where its sensitivity is significantly greater than that of bpMRI (22). Additionally, the enhanced imaging capabilities of mpMRI facilitate more precise prostate cancer localization, thereby reducing the false-positive rate (33). When the mpMRI result is negative, the negative predictive value for significant prostate cancer is as high as 95% (40). In AS, mpMRI can detect tumor progression (such as a volume change of ≥ 50%) at an earlier stage and guide the appropriate timing of treatment (41).

PI-RADS, through the interpretation of standard mpMRI images, has significantly enhanced the detection efficiency of csPCa. Research has validated that PI-RADS 4–5 lesions are strongly correlated with csPCa, with positive predictive values (PPV) of up to 48.1% and 68.3% respectively (42, 43). However, the clinical management of PI-RADS 3 lesions remains a subject of controversy, with a csPCa detection rate of only 12.5%-20.8%. Individualized decisions should be made by incorporating high-risk factors such as prostate-specific antigen density (PSAD > 0.15 ng/ml/cm³) or abnormal digital rectal examination (44, 45). It is important to note that even though PI-RADS 5 lesions have a high predictive value, 18% of cases yield benign pathological results following targeted biopsy, suggesting that integration of other imaging or molecular markers is necessary to optimize diagnostic efficacy (46, 47). In response to the clinical challenges posed by PI-RADS 3 lesions, the latest guidelines advocate a dynamic risk-stratification strategy: for single-focus lesions with a low PSAD (PSAD < 0.12 ng/ml/cm³), short-term imaging follow-up can be implemented, while for multiple-focus lesions or those with high-risk factors, MRI-ultrasound fusion-guided targeted biopsy is recommended (44, 45, 48). Moreover, elastography technology, by quantifying tissue hardness disparities, can assist in locating sclerotic areas not visualized by MRI. When combined with mpMRI, it can increase the csPCa detection rate by 8%-12%, particularly providing supplementary value in transition-zone lesions (45, 46).

### 3.3 Molecular imaging breakthrough of PSMA PET/CT

#### 3.3.1 Precise targeting and tumor heterogeneity assessment of PSMA PET/CT

PSMA PET/CT enables highly sensitive detection (sensitivity 90-98%, specificity 82%-99%) of prostate cancer lesions by targeting the expression of the PSMA protein (49, 50). It can also furnish information regarding the body-wide distribution of tumors, assisting doctors in comprehensively evaluating disease spread and thereby formulating more precise treatment plans (51, 52). Studies have indicated that PSMA PET/CT demonstrates high sensitivity and specificity in identifying local and distant metastases (49, 53), particularly in high-risk prostate cancer patients, where its diagnostic accuracy surpasses that of traditional imaging methods (CT and MRI) (54). The maximum standardized uptake value (SUVmax) of PSMA PET/CT is significantly positively correlated with tumor aggressiveness (such as Gleason score, pathological stage) (p=0.007) (55, 56). In instances where mpMRI results are negative or equivocal, PSMA PET/CT can function as an effective supplementary tool.

#### 3.3.2 Challenges of PSMA-negative tumors and exploration of new targets

Approximately 5%-10% of prostate cancers (notably those with neuroendocrine differentiation) display low PSMA expression (SUVmax < 10), and a missed diagnosis could potentially delay treatment (57, 58). For such cases, alternative biomarkers like KLF8, CHST11, or functional imaging (such as FDG-PET) must be incorporated (56, 57). Research reveals that the combined use of mpMRI and PSMA PET can elevate the negative predictive value (NPV) of biopsy to 96%, diminishing the necessity for systematic biopsy (58). The detection rate of PSMA-PET for tumors with a high Gleason score (≥8) is markedly higher than that for tumors with a low score (90% vs 60%), yet its sensitivity to Gleason 6 tumors is inadequate (<50%) (57, 58). Additionally, antibody probes targeting KLF8 have manifested potential for specific binding to poorly differentiated tumors in preclinical studies, and future endeavors are required to facilitate their clinical translation (57).

## 4 AI-driven multimodal image fusion and target region identification

### 4.1 Artificial intelligence and image analysis technologies

#### 4.1.1 Lesion segmentation based on deep learning

AI, leveraging deep-learning algorithms, has empowered automated analysis of prostate imaging data and extraction of quantitative features, thereby significantly enhancing the accuracy and efficiency of lesion localization. AI models, such as U-Net and nnUNet, are capable of automatically segmenting prostate anatomical structures and suspicious lesions by analyzing mpMRI and PET images (59). For instance, FocalNet, through the integration of convolutional neural network and Gleason score data, has accomplished joint detection of prostate cancer and prediction of its aggressiveness. Its sensitivity and specificity in the detection of clinically significant prostate cancer attain 89.7% and 87.9% respectively (60). In contrast, traditional image segmentation hinges on manual delineation, which is not only time-consuming but also prone to subjective bias. Deep-learning models can mitigate the subjective discrepancies among radiologists and enhance the consistency of segmentation results. For example, a study encompassing 976 cases demonstrated that the consistency between the AI segmentation model based on ADC maps and the manual annotations of multiple radiologists reached 0.96 (95% CI 0.95-0.97) (61).

#### 4.1.2 Quantification of image texture features

Subsequent to lesion segmentation, the technique of image texture feature quantification can be further employed to conduct a more in-depth analysis of the segmented lesion area. Texture feature quantification has the capacity to capture subtle alterations in the lesion area, such as cell arrangement and blood vessel distribution. This information aids in the evaluation of the malignancy and aggressiveness of the lesion. For example, regarding PI-RADS 3 lesions, the AI classification model founded on the texture features of T2-weighted images exhibits a sensitivity and specificity of 83% and 96% respectively for csPCa, markedly outperforming traditional visual assessment (Area Under Curve [AUC] 0.89 vs 0.72, p < 0.001) (62). Three-dimensional morphological analysis techniques, like light-sheet microscopy, further augment the model’s ability to capture heterogeneous structures and enhance diagnostic robustness (63).

### 4.2 Multimodal image fusion

Multimodal image fusion technology significantly enhances the precision and efficiency of prostate biopsy by integrating the complementary advantages of MRI, US, and CT.

#### 4.2.1 MRI-TRUS fusion

By integrating the high-resolution anatomical information of MRI with the real-time navigation functionality of ultrasound, a three-dimensional model is constructed to facilitate puncture positioning. Targeted biopsy using MRI-TRUS fusion in conjunction with systematic biopsy (TB + SB) can significantly boost the detection rate of csPCa. Compared to traditional 12-core TRUS-Bx, fusion biopsy has demonstrated superiority in large-scale clinical trials such as PRECISION and PRECISE, particularly in the detection of high-risk tumors with an International Society of Urological Pathology (ISUP) grade of ≥ 2 (16–18). Prospective multicenter trials have revealed that mpMRI combined with TRUS fusion biopsy yields a significantly higher csPCa detection rate than simple systematic biopsy. The biopsy positive rate is increased by 20%-30%, while the overdiagnosis of low-risk tumors is reduced (64–66). In comparison to the transrectal approach, transperineal MRI-TRUS fusion biopsy exhibits a lower infection complication rate (0.8% vs. 3.5%) (2, 67), and antibiotic use is more standardized. Although pain and anxiety persist in fusion biopsy, advancements in electromagnetic tracking and local anesthesia techniques (such as the Vector electromagnetic needle tracking system) have improved patient tolerance (68, 69). A comparative analysis of different fusion methods of MRI - TRUS is presented in **Table 2**.

**Table 2:** Comparative analysis of different MRI-TRUS fusion methods.

Dimension	Cognitive fusion	Real-time fusion	AI-driven fusion
Precision	Moderate, contingent on operator experience	High (error < 3 mm)	High (error < 1 mm)
Efficiency	Time-consuming, requiring manual registration	Rapid, with automatic registration	Extremely rapid, leveraging AI-automated path optimization
Cost	Low	Medium	High
Application Scenarios	Primary-level hospitals or clinicians with substantial experience	Hospitals of medium to large scale	Large -scale hospitals or technology demonstration centers

#### 4.2.2 Exploration of fusion of other imaging technologies

PSMA-PET/CT and Ultrasound Fusion: PSMA-PET/CT can specifically identify the molecularly metabolically active regions of prostate cancer lesions, while ultrasound offers real-time anatomical guidance. The integration of these two modalities combines highly sensitive molecular imaging with real-time puncture navigation, significantly increasing the detection rate of csPCa (70–72). PSMA-PET/CT can also pinpoint multifocal or occult lesions, thereby avoiding blind punctures in non-metabolically active areas. For instance, in PSMA-PET-negative areas, the csPCa missed diagnosis rate is merely 5%-8% (25, 73). whereas the missed diagnosis rate of TRUS-Bx is as high as 20%-30% (71).

PET and MRI Fusion: The PET-MRI fusion technology combines the functional metabolic information of PET with the anatomical structure information of MRI, surmounting the limitations of single-technology approaches. By simultaneously acquiring metabolic and anatomical data, doctors can more comprehensively assess the location, size, and activity of lesions. For example, PSMA-PET/MRI can identify metabolically active lesions that MRI might overlook, and MRI can supply anatomical details to aid in targeted biopsy (74, 75).

### 4.3 Target region identification

In cognitive fusion, doctors visually incorporate the information of suspicious lesions in mpMRI images (such as those with a PI-RADS score ≥ 3), subjectively localize them under the guidance of TRUS, and subsequently direct the biopsy. Cognitive fusion does not necessitate special software or equipment; rather, it only requires a conventional TRUS biopsy system (17, 76). This approach circumvents the costs associated with developing or procuring complex image fusion algorithms (77, 78). Nevertheless, there are substantial disparities among different physicians in the identification and spatial registration of MRI lesions, leading to low detection consistency (77, 79). If the surgeon has not undergone professional MRI image-reading training or solely relies on the imaging report without independently assessing the images, inexperienced doctors may overlook small lesions or misinterpret the lesion boundaries, significantly augmenting the risk of biopsy errors (76, 78). Studies have indicated that approximately 5%-13% of csPCa might be undetected in cognitive targeted biopsies, and systematic biopsy must be combined to enhance sensitivity (69, 80).

**Figure 1:**
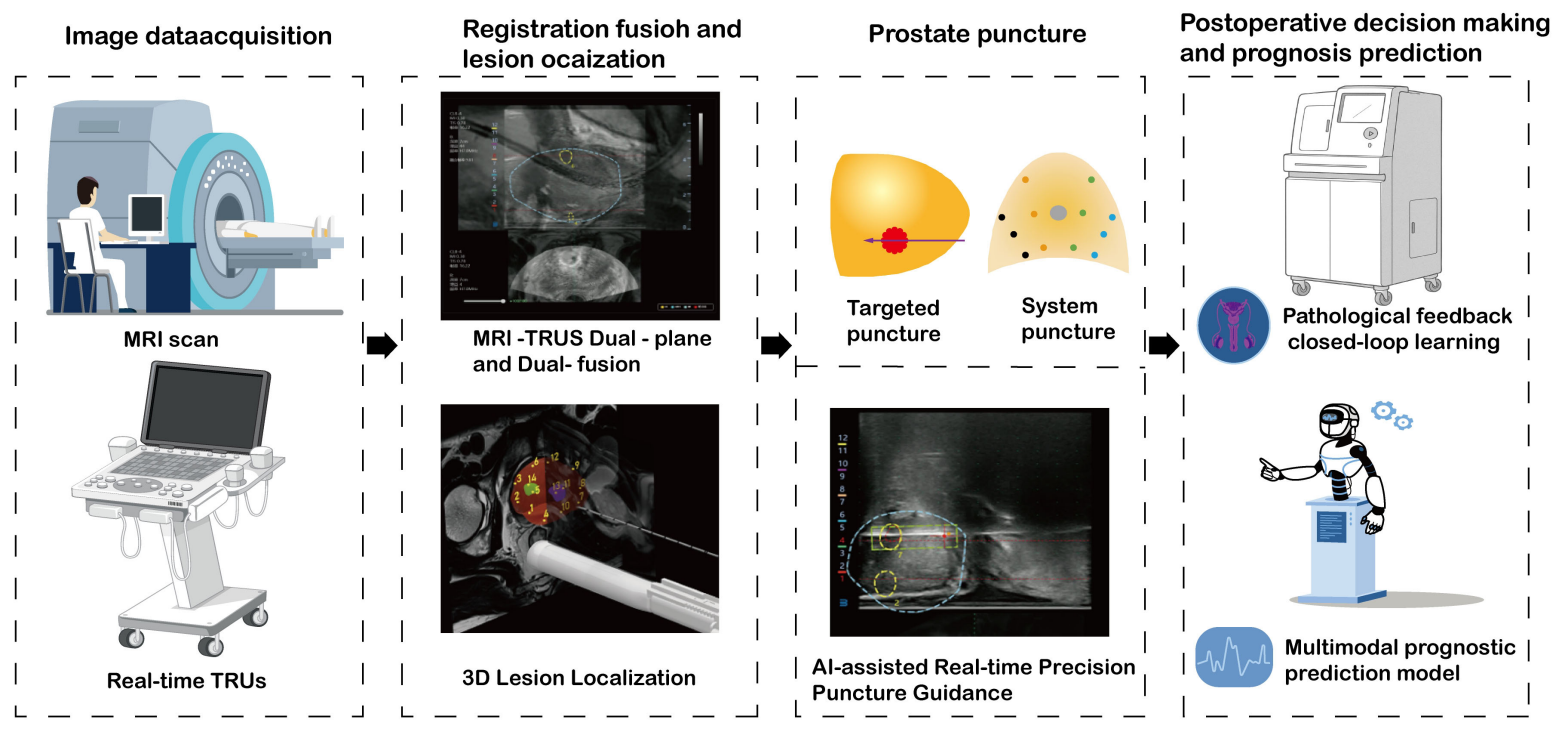
A comprehensive workflow of artificial intelligence-assisted prostate biopsy: from image acquisition to prognosis prediction.

AI software fusion: Deep-learning algorithms, like convolutional neural networks, are utilized to automatically delineate the prostate region and lesions within mpMRI or bpMRI. Coupled with elastic registration technology, the MRI and TRUS images are precisely merged to accomplish automatic target-region marking and navigation. AI algorithms mitigate the variability of human interpretation, particularly demonstrating stable performance in multi-center and multi-scanner scenarios (79, 81). Automated segmentation and registration decrease the reliance on physician experience and shorten the learning curve (82, 83). AI models can optimize the interpretation of bpMRI (T2WI + DWI) to reduce costs while sustaining accuracy. For instance, the bpMRI AI model maintained a PPV comparable to that of mpMRI in external validation (79, 84). Compared to cognitive fusion technology, targeted biopsy guided by AI software (such as Biopsee, UroNav) elevates the csPCa detection rate by approximately 10%-15% and exhibits a greater capacity to distinguish PI-RADS 3-score lesions (77, 85, 86). AI software fusion is characterized by high precision and a low learning threshold, yet the equipment cost is relatively high, rendering it suitable for centers that prioritize standardized diagnosis (82, 87). In the foreseeable future, AI-assisted prostate biopsy will realize a fully automated closed-loop process encompassing pre-operative, intra-operative, and post-operative phases. Specifically, during the pre-operative phase, state-of-the-art AI algorithms will automatically analyze DICOM imaging data to generate a personalized biopsy plan. This plan will specify the optimal number of needles, precise puncture paths, and potential risk warnings based on anatomical landmarks and lesion localization. In the intra-operative stage, a robotic system will execute the biopsy procedure, whereas AI-driven real - time tracking will compensate for spatial deviations caused by respiratory motion or patient body movements. This will ensure millimeter-level accuracy through dynamic path correction algorithms. Post-procedure, the system will autonomously generate a comprehensive structured clinical report that integrates histopathological findings with quantitative imaging biomarkers. Supported by AI-powered analysis, the report will recommend patient-specific follow-up intervals aligned with clinical guidelines. This end-to-end automation framework aims to standardize biopsy workflows, minimize operator dependency, enhance diagnostic consistency in prostate cancer management, thereby improving the overall quality of prostate cancer diagnosis and treatment. As depicted in **Figure 1**, a schematic diagram illustrates the specific biopsy procedure in detail.

### 4.4 Limitations and challenges of AI in prostate biopsy

Despite promising results, the widespread adoption of AI-driven biopsy systems faces several challenges:

Generalizability: AI models trained on single-center data may underperform in external validation due to variations in MRI scanners, protocols, and patient populations (88). Data Dependency: High-quality annotated datasets are scarce, limiting the development of robust models (89, 90). Cost and Accessibility: AI software and robotic biopsy systems require significant investment, hindering deployment in resource-limited settings. Future efforts should focus on multi-center collaborations, federated learning, and cost-effective AI solutions to enhance accessibility (89, 91).

## 5 Conclusion

The collaborative utilization of imaging technologies and artificial intelligence has substantially enhanced the precision of prostate cancer biopsy and diagnosis. However, these precision diagnostic tools directly influence treatment decisions, such as qualifying patients for active surveillance or guiding focal therapy. The combination of MRI-TURS targeted biopsy and systematic biopsy can effectively lower the missed-diagnosis rate. Meanwhile, PSMA PET/CT compensates for the limitations of traditional imaging by providing molecular metabolic information. AI technology, via automated segmentation, texture-feature quantification, and multimodal data fusion, has decreased operator dependence. Nevertheless, its clinical dissemination is constrained by equipment costs and the generalization ability of algorithms. Future research directions should center on:

1. Technology Integration and Standardization: Promote the in-depth integration of multimodal imaging (MRI, PET, ultrasound) with genomic data to construct individualized risk-prediction models. Develop cross-platform AI algorithms and enhance the robustness and clinical applicability of these models through multi-center validation.
2. Optimization of Precise Diagnosis: For PI-RADS 3 lesions, create dynamic risk-stratification AI tools and achieve precise differentiation by integrating molecular markers and radiomic features. Explore novel molecular probes for PSMA-negative tumors and multimodal imaging complementary strategies.
3. Clinical Translation and Accessibility: Simplify the AI-assisted diagnosis procedure and reduce hardware reliance. Promote the low-cost screening approach of bpMRI combined with AI to facilitate the dissemination of this technology in resource-constrained regions.

Through interdisciplinary collaboration and technological evolution, the collaborative application of imaging and AI will propel the diagnosis and treatment of prostate cancer toward intelligent whole-process management, ultimately leading to a comprehensive improvement in patient prognosis and efficient utilization of medical resources.
